# Salicylic Acid as an Adulteration

**Published:** 1881-07

**Authors:** 


Salicylic Acid as an Adulteration.—In February last a ministerial circular was issued, prohibiting the sale of any food or drink which contained any salycilic acid. This action was taken in consequence of representation made by M. Vallin, who stated that such large quantities of the acid were introduced into wine and beer as to be dangerous. Thus M. Vallin asserts that from fifteen to fifty-two grains of the acid to a quart of beer were found. At this rate, we are told that a French laborer is liable to take fifty or sixty grains of the acid in a day. Such a quantity is, he says, far from being innocent. We should say, however, that if the average French laborer can stand a gallon of beer a day, he will not be made much worse by a little salycilic acid.

The *Cincinnati Lancet and Clinic* states that some contrary investigations have been made by Dr. Galippe, who shows that the evils from salycilic, especially that of its producing anaphrodia, are not so great as is asserted.—*Medical Record.*

